# Correction: Thorium amidates function as single-source molecular precursors for thorium dioxide

**DOI:** 10.1039/d4cc90297a

**Published:** 2024-09-06

**Authors:** Mark D. Straub, Erik T. Ouellette, Michael A. Boreen, Jacob A. Branson, Alex Ditter, A. L. David Kilcoyne, Trevor D. Lohrey, Matthew A. Marcus, Maria Paley, José Ramirez, David K. Shuh, Stefan G. Minasian, John Arnold

**Affiliations:** a University of California, Berkeley Berkeley CA 94720 USA arnold@berkeley.edu; b Lawrence Berkeley National Laboratory Berkeley CA 94720 USA sgminasian@lbl.gov

## Abstract

Correction for ‘Thorium amidates function as single-source molecular precursors for thorium dioxide’ by Mark D. Straub *et al.*, *Chem. Commun.*, 2021, **57**, 4954–4957, https://doi.org/10.1039/D1CC00867F.

The single crystal X-ray crystallographic data for Th(ITA)_4_ (ITA = *N-tert*-butylisobutyramidate) has been reanalysed using a *P*2_1_/*n* unit cell. The structure of Th(ITA)_4_ displayed full-molecule disorder when solved in the space group *C*2/*m*, as originally reported. However, revisiting the same single crystal X-ray crystallographic data showed a weaker *P*2_1_/*n* supercell containing an ordered arrangement of the ligands. The supercell was overlooked previously due to the strength of the heavy atoms in the crystal. The revised X-ray crystal structure of Th(ITA)_4_ is shown in Fig. 1, with associated bond metrics and crystallographic data given in Tables 1 and 2. This change to the model of the X-ray structural data does not impact any other aspects of the data or conclusions reported previously.

**Fig. 1:**
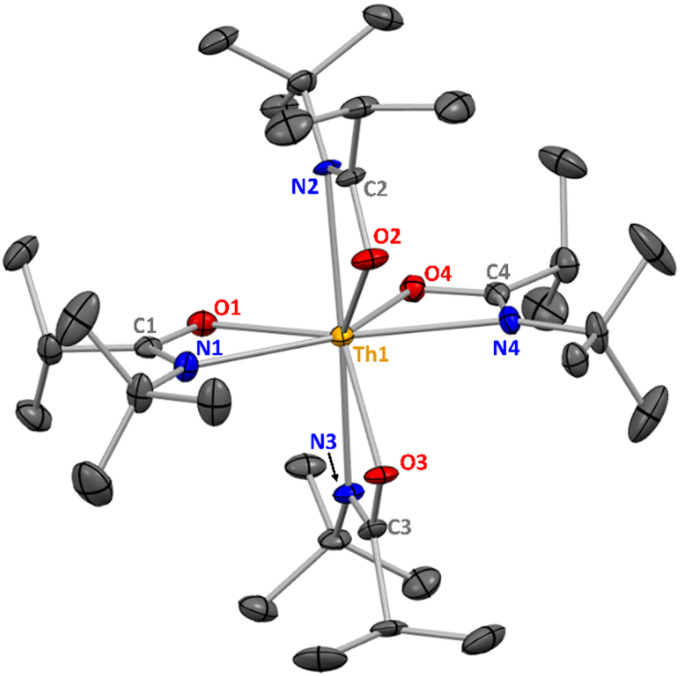
X-ray crystal structure of **1** with 50% probability ellipsoids. Hydrogen atoms and structural disorder are omitted for clarity.

**Table 1:** Selected atomic distances (Å) and angles (°) for Th(ITA)_4_

Th1–O1	2.361(3)	C3–O3	1.305(6)
Th1–O2	2.416(3)	C4–O4	1.308(5)
Th1–O3	2.424(3)	C1–N1	1.299(5)
Th1–O4	2.356(3)	C2–N2	1.309(7)
Th1–N1	2.565(4)	C3–N3	1.310(7)
Th1–N2	2.557(5)	C4–N4	1.312(6)
Th1–N3	2.557(5)	O1–C1–N1	115.9(4)
Th1–N4	2.536(4)	O2–C2–N2	116.0(5)
C1–O1	1.312(5)	O3–C3–N3	116.7(5)
C2–O2	1.306(6)	O4–C4–N4	115.6(4)

**Table 2:** Crystallographic details and refinement metrics for Th(ITA)_4_

Chemical formula	C_32_H_64_N_4_O_4_Th
Formula weight	800.91
Color, habit	Colorless, block
Crystal system	Monoclinic
Space group	*P*2_1_/*n*
*a* (Å)	8.8073(4)
*b* (Å)	24.7927(11)
*c* (Å)	17.2472(7)
*α* (°)	90
*β* (°)	94.9595(16)
*γ* (°)	90
*V* (Å^3^)	3751.9(3)
*Z*	4
Density (g cm^−3^)	1.418
*F*(000)	1624.0
Radiation type	Synchrotron
Radiation wavelength	(*λ* = 0.7288 Å)
*μ* (mm^−1^)	4.275
Crystal size (mm)	0.18 × 0.10 × 0.09
Meas. refl.	11 511
Indep. refl.	6998
Obsvd. [*I* > 2*σ*(*I*)] refl.	6998
*R*_int_	0.0422
Final [*I* ≥ 2*σ*(*I*)] *R* indices	*R*_1_ = 0.0313
	w*R*_2_ = 0.0660
Goodness-of-fit	1.047
CCDC	2377153

The Royal Society of Chemistry apologises for these errors and any consequent inconvenience to authors and readers.

